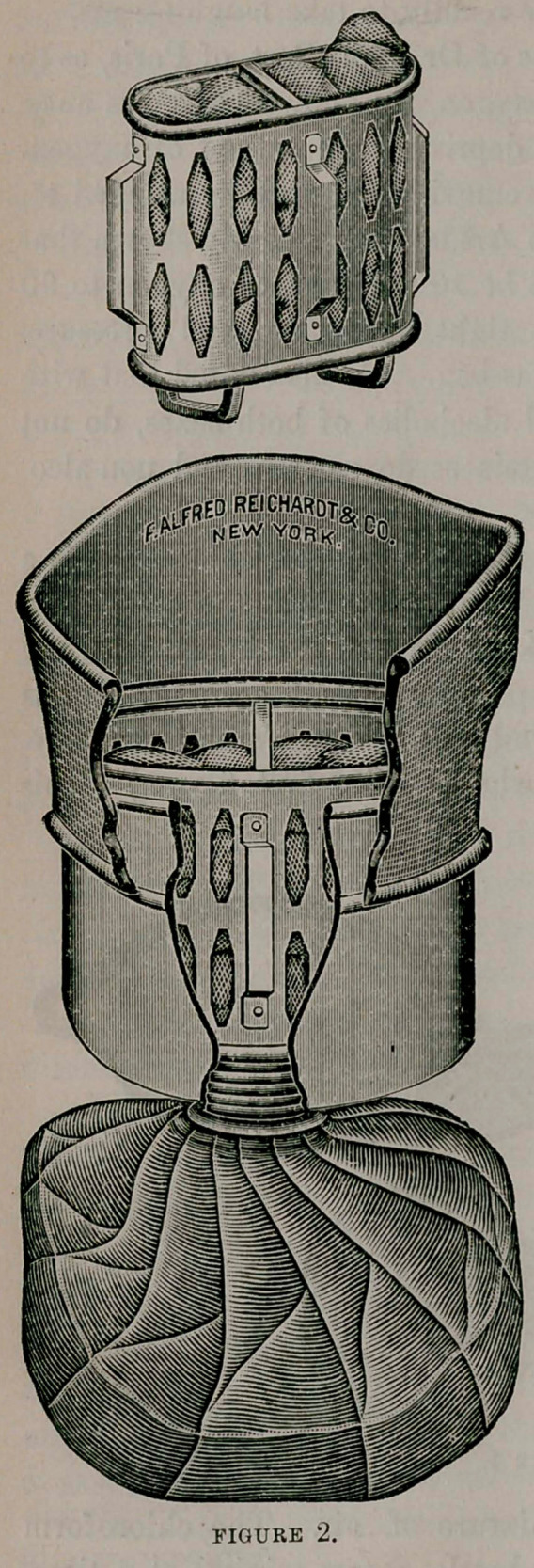# General Considerations upon Major Anesthesia

**Published:** 1897-08

**Authors:** Robert H. M. Dawbarn

**Affiliations:** Professor of Surgery and of Surgical Anatomy, New York Polyclinic College and Hospital


### ATLANTA
### Medical and Surgical Journal.

VOL. XIV. AUGUST, 1897. No. 6.

L. B. GRANDY, M.D., MANAGING EDITOR.
M. B. HUTCHINS, M.D., BUSINESS MANAGER.

COLLABORATORS:
A. W. CALHOUN, M.D., LL.D., VIRGIL O. HARDON, M.D., FLOYD W. McRAE, M.D., A. W. STIRLING, M.D., JOSEPH EVE ALLEN, M.D., AUGUSTA, GA., AND HENRY R. SLACK, PH.M., M.D., LAGRANGE, GA.

### ORIGINAL COMMUNICATIONS.

### GENERAL CONSIDERATIONS UPON MAJOR ANESTHESIA.

By ROBERT H. M. DAWBARN, M.D.,

Professor of Surgery and of Surgical Anatomy, New York Polyclinic College and Hospital.

Which shall we use—ether or chloroform? There is no study less profitable than that of the relative death-percentages all over the world from chloroform *vs.* ether. It is absolutely wasted time. That von Nussbaum saw chloroform used forty thousand times without a death,* and that chloroform has been exhibited thirty-six thousand five hundred times in the Edinburgh Infirmary with but one fatal result,† would seem evidence that such danger from the use of this anesthetic as exists is largely due to the personal equa-

*Wood's Reference Handbook, Vol. I., p. 189.

†*American Journal of Medical Sciences*, November, 1888.

tion; that is, to inexperience on the part of the anesthetizer, or to improper methods of meeting the emergency.

In New York, Philadelphia and Boston there is a curious distrust of chloroform not apparent in the rest of the world of surgery to the same degree, either abroad or in this country.

The writer was one of a hundred or more auditors when that famous surgeon, Dr. Henry B. Sands, once told a story which it required a man of his eminence and his truthfulness to relate, concerning an experience of his in Germany. He was present during an operation by Schede in the *Allgemeines Krankenhaus* in Hamburg-Eppendorf, when Schede happened to remark that he had never in his life seen ether given; that he and all the famous men of his country relied solely upon chloroform. Dr. Sands thereupon agreed to enlighten his ignorance, and taking the anesthetist's place gave ether to the next patient. It is painful to relate that this patient had the bad taste to die from the ether, thus casting discredit upon a most valuable drug. Dr. Sands said that he and Dr. Schede worked over the man with artificial respiration and other means for a long while, but failed to revive him. And I venture to say that Dr. Schede now has the same distrust and fear of ether as an anesthetic which seems to prevail regarding chloroform in the trinity of cities just named.

It is admitted that, taking averages, more deaths on the table occur from chloroform than from ether; but if we were to add those happening weeks and months later, due to ether-irritation of lungs or kidneys in persons already enfeebled in these organs, perhaps the percentages would be different.

It is unquestionably true that more skill is needed to give chloroform safely than ether. Were the writer to need an operation upon himself he would choose ether, if subjected to an "emergency" anesthetizer, but chloroform if that assistant were skilled and experienced.

Even in the three cities just mentioned together, however, certain conditions are recognized as justifying the use of chloroform as against ether. These are:

1. The presence of severe pain. Chloroform is relatively safe in childbirth, even in the hands of inexperience. The reason is unknown.*

2. In childhood; at least, this is the teaching of Dr. Jacobi and of numerous other children's specialists.

3. To control convulsions—uremic, epileptic, strychnic *et al.* Here ether would be too slow in taking effect.

4. In cases where actual cautery must be used about the mouth, ether being inflammable.

5. In certain diseases of the lungs, kidneys, stomach, brain.

6. In military surgery, being less bulky, more rapid in action, and cheaper.

7. In hot climates (ether boils at 95 degrees F., chloroform at 142 degrees F.)

8. At night, if a flame must be held near the patient. (But chloroform is not free from disadvantage at night, being decomposed by the flame, and chlorine and other gaseous irritants set free. All surgeons have noticed the tendency to cough under such circumstances.)

*The Question of Mixtures.*—This is one that remains unsettled, the widest divergence of views continuing. Dr. J. C. Reeve, already quoted as to relative mortality, says: "The chloroform committee of the Medico-Chirurgical Society of London, in 1864, recommended, among others, a mixture of one part alcohol, two parts chloroform and three parts ether, by measure, known as the 'A. C. E. mixture,' which has probably been used more than any other. At Vienna a mixture of six parts of ether to one of chloroform has been used so much as to be known as the 'Vienna mixture.' It is stated that there have been eight thousand administrations of it without a death. Billroth, of the same city, used a mixture of three parts of chloroform, one of ether, and one of alcohol."

Against such mixtures the argument has always been urged that

---

*H. C. Wood says (Therapeutics, 7th edition, p. 150): "So far as I know, no death has yet occurred from chloroform during parturition."

Wood's Reference Handbook, Vol. I., p. 195: "Chloroform has been used in natural labor many hundreds of thousands of times, yet but a single case of death is on record where it was administered by a competent medical man, and in this instance there is lack of post-mortem confirmation."

a new chemical is not thus made, with a single rate of diffusion; and that while the rate of relative evaporation is doubtless in part modified by the mixture, the patient substantially gets first ether, then chloroform, then alcohol, in order of volatility. The writer agrees with the majority of surgeons in feeling that a gain in security is not made thereby. Straight drinks are safest—to speak after the manner of the world.

*Chloride of Methylene* ($CH_2Cl_2$).—This is mentioned simply as standing as a type of drugs belonging more or less to the chloroform class—the halogen anesthetics. This short essay does not permit of a careful discussion of them. This particular one enjoys the indorsement of Sir Spencer Wells, who, after considerable use of it, regards it as the best anesthetic.

*Nitrous Oxide.*—The writer believes that laughing-gas will be considerably more used in the future, in general surgery, than in the past. Now that it is obtainable in liquid form in small steel canisters of such a size that one may be slipped within a small handbag, the main objection to its use has disappeared.* A special closely fitting mask and a large thin rubber bag to hold the gas generated from the liquid complete the outfit.

Heretofore its exhibition has remained almost solely in the hands of dentists; but it would seem that there is a field here for young physicians to fill. The writer has many times employed a certain dentist to give gas at the patient's house, and on more than one occasion the operation has lasted, with satisfactory anesthesia, fully three-quarters of an hour.

As every one knows, the color of the patient meanwhile is always ghastly in its lividity; but this seems not dangerous in realty. Differing from ether or chloroform, should alarming symptoms appear, removal of the inhaler is followed by prompt improvement almost with the first breath of pure air inhaled. Of course some little skill is needed to keep the patient from waking repeatedly during an operation of some length, but it is certain that the experience and the care need not be more than for the safe use of chloroform. It is a pleasure to see the absence of the initial choking and strug-

*It is liquified by about fifty atmospheres; and 600 pounds pressure to the cubic inch are necessary to keep it so.

gling, and of subsequent anorexia and nausea with these patients, and certain ones who would refuse a needed operation, because of the ether or chloroform, will agree readily to take laughing-gas.

Due largely to the investigations of Dr. Paul Bert, of Paris, as to exhibition of nitrous oxide with oxygen, we know that it has anesthetic qualities aside from merely depriving the patient of oxygen. And the addition of oxygen, by removing the lividity alluded to, seems desirable. Dr. W. W. Van Arsdale has recently shown that the best proportion would seem to be 10 per cent. of oxygen to 90 per cent. of the $N_2O$—with a tight face-mask, and pressure, as from a heavy book, upon the gas-bag. He also noted that with this addition of oxygen, men, and alcoholics of both sexes, do not so readily succumb to the anesthesia as do women, and non-alcoholic subjects.

The kind of inhaler to be used for ether and for chloroform is a matter of no little importance. With chloroform the safest proportion is estimated at from 3 to 4 per cent. of it mingled with the inspired air. Clover's apparatus permits of such accuracy, but is very expensive. In practice we find that Esmarch's inhaler is safe, and it is the one most used everywhere. (See first figure.) This

FIGURE 1.

device permits of abundant admixture of air. The chloroform should be poured on from a drop-bottle, a few minims at a time.

The improved Esmarch inhaler shown in the illustration allows instant change of the flannel or gauze cover, for each new patient—a decided gain in cleanliness. In use, the covering material is laid upon the wire frame, over this the wire rim is snapped into place, and any excess of the covering is trimmed away with scissors.

FIGURE 2.

As to ether-inhalers upon the market, their number is considerable. Perhaps, however, the familiar towel-and-newspaper cone of home manufacture is still the favorite with the bulk of the profession. It has at least the merit of cleanliness, for the same cone is seldom used twice. Still, with ether quoted at upwards of a dollar a pound, and the ordinary estimate being a pound for each hour of anesthesia, it is plain that the device is an expensive one.

Allis's inhaler has been used to an enormous extent, and need not be described. It also is wasteful of ether, and as it is rather bothersome to remove the old and replace by new the numerous folds of bandage on which the ether is poured, too often we see these inhalers, though soiled by sputum and other ejecta, continued for some time in use upon successive patients.

The ether-inhaler figured herein (second figure) was devised by the writer some ten years ago, and has since been continually used at his clinic and in private practice. A few years ago Dr. F. H. Wiggin, gynecologist to the New York City Hospital, wrote a commendatory article upon it after using it for some time. It is made by F. A. Reichardt & Co., of 27 Barclay street, this city, to whom the author feels indebted for the ingenuity and painstaking care which have so improved upon the original suggestion.

Its advantages are (*a*) economy, and (*b*) ease of cleansing, as well as (*c*) those benefits and added safety to the patient resulting from avoidance of saturation of all his tissues by a great excess of ether. The apparatus is upon the same principle as the well-known one of Clover, but is less complicated and less expensive.

(*a*) As to economy, the thing will pay for itself, in saving of ether after a very few long operations. It is literally true that only one-fourth to one-fifth as much ether is needed as with a home-made cone; indeed, hardly more of ether than one uses of chloroform in an equally long operation, provided a skilled anesthetizer is in charge. Also, it is simple and durable, and should last for years.

(*b*) The stout open cage of nickel-plated metal fitting within the solid outer frame receives a little gauze or cotton, or a handkerchief. On this the ether is poured. At the end of the operation the cage is removed, emptied, held beneath the hot water faucet for a moment, and it is clean and ready for the next case.

The face-piece of stout rubber of course is removable for washing, as is the air-bag. This latter is a light, thin rubber bag, of a capacity considerably greater than that of the lungs. The solid rubber face-piece is more durable and simpler than are the pneumatic ones seen on certain other inhalers, and fits the face quite closely enough. To avoid one more contrivance demanding cleansing, no device is used (as with the Clover and the Ormsby, for instance) for pouring in ether without removal from the face. In my inhaler, just as with the home-made cone, when more ether is needed the cone is removed long enough—a few seconds only—to receive it.

(*c*) Under this heading the reader will observe that because of the air-bag attachment the ether is rebreathed again and again.

This, of course, economizes ether, but what is more important, it saves surcharging the blood, and the lungs, kidneys and other viscera. The ether thus warmed is also, because of that warming, less apt to cause an ether-pneumonia; continued evaporation of ether being very chilling, as every one knows. Since much less ether is given, it follows that the danger of death from ether-narcosis is lessened. It is surprising to one accustomed to the home-made cone to see how quickly the patient recovers entire consciousness; whereas by the former device the breath remains redolent of ether for about twenty-four hours, and for the same reason the anorexia is long continued; by the use of the inhaler I am discussing the elimination is completed and the patient's appetite has returned in much less time.

Because of rebreathing the same air a $CO_2$ anesthesia is superadded to that from the ether, and consequently the patient is almost as quickly anesthetized and with as little struggling as if from chloroform. To the writer this seems one of the distinct advantages of the Clover air-bag principle, and he would differ from those who consider the $CO_2$ an objection.* It is, of course, easy to observe the color of the skin or mucous membranes, and if more oxygen is desired, to hold the cone less tightly to the face, permitting at any time an admixture of air.

In the current April and June numbers of the New York *Poly-clinic Journal* the writer has further discussed general anesthesia, with especial reference to the best means of combating threatened death from this cause.†

(*To be continued.*)

---

Sties may sometimes be aborted by an inunction of the yellow oxide of mercury, or by the application of a saturated solution of boracic acid. Hot compresses relieve the pain. When sties appear at frequent intervals, the internal use of sulphuret of calcium is recommended.—*Hare.*

*See in corroboration of this view several experiments quoted in Wood's Therapeutics, 7th edition, p. 135, showing that a carbonic acid anesthesia is a safe one.

†These articles are now being revised and amplified by the author for publication in THE ATLANTA MEDICAL AND SURGICAL JOURNAL.

## Figures and Tables

**FIGURE 1. f1:**
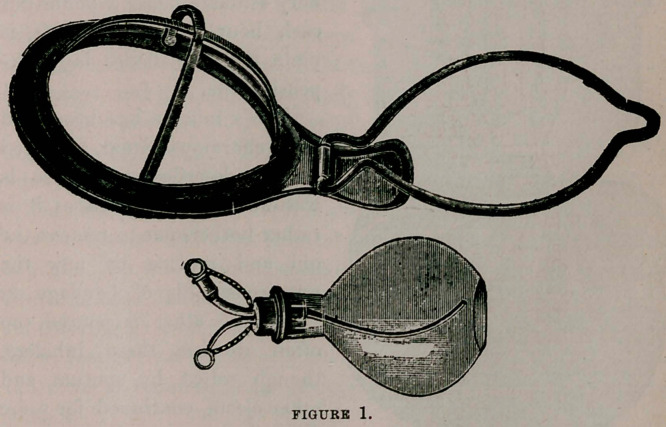


**FIGURE 2. f2:**